# Detection of mRNA Transcript Variants

**DOI:** 10.3390/genes16030343

**Published:** 2025-03-16

**Authors:** Kevin Vo, Sharmin Shila, Yashica Sharma, Grace J. Pei, Cinthia Y. Rosales, Vinesh Dahiya, Patrick E. Fields, M. A. Karim Rumi

**Affiliations:** Department of Pathology and Laboratory Medicine, University of Kansas Medical Center, Kansas City, KS 66160, USA; kvo5@kumc.edu (K.V.); sharminshila.mib@gmail.com (S.S.); yashica2025@gmail.com (Y.S.); 3096823@smsd.org (G.J.P.); 255cdy30@students.olatheschools.com (C.Y.R.); vinesh.dahiyampharm@gmail.com (V.D.); pfields@kumc.edu (P.E.F.)

**Keywords:** gene expression, mRNA transcript variants, RNA sequencing, spatial transcriptomics, analyses of NGS data, verification of NGS data

## Abstract

Most eukaryotic genes express more than one mature mRNA, defined as transcript variants. This complex phenomenon arises from various mechanisms, such as using alternative transcription start sites and alternative post-transcriptional processing events. The resulting transcript variants can lead to synthesizing proteins that possess distinct functional domains or may even generate noncoding RNAs, each with unique roles in cellular processes. The generation of these transcript variants is not merely a random occurrence; it is cell-type specific and varies with developmental stages, aging processes, or pathogenesis of diseases. This highlights the biological significance of transcript variants in regulating gene expression and their potential impact on cellular functionality. Despite the biological importance, investigating transcript variants has been hampered by challenges associated with detecting their expression. This review article addresses the advancements in molecular techniques in detecting transcript variants. Traditional methods such as RT-PCR and RT-qPCR can easily detect known transcript variants using primers that target unique exons associated with the variants. Other techniques like RACE-PCR and hybridization-based methods, including Northern blotting, RNase protection assays, and microarrays, have also been utilized to detect transcript variants. Nevertheless, RNA sequencing (RNA-Seq) has emerged as a powerful technique for identifying transcript variants, especially those with previously unknown sequences. The effectiveness of RNA sequencing in transcript variant detection depends on the specific sequencing approach and the precision of data analysis. By understanding the strengths and weaknesses of each laboratory technique, researchers can develop more effective strategies for detecting mRNA transcript variants. This ability will be crucial for our comprehensive understanding of gene regulation and the implications of transcript diversity in various biological contexts.

## 1. Introduction

Mammalian cells are versatile and complex in expressing various types of ribonucleic acids (RNAs), each transcribed by distinct RNA polymerases that play essential roles in cellular function. Specifically, RNA polymerase I (Pol I) transcribes ribosomal RNAs, which are fundamental components of the ribosome and necessary for protein synthesis. RNA polymerase II (Pol II), on the other hand, transcribes precursor messenger RNAs (pre-mRNAs), which serve as templates for protein-coding sequences. Meanwhile, RNA polymerase III (Pol III) transcribes transfer RNAs (tRNAs) and a variety of other small RNAs that are crucial for various cellular processes [1]. While a subset of pre-mRNAs is processed into mature protein-coding mRNAs, a significant proportion of the transcripts generated by RNA polymerases I, II, and III are classified as noncoding RNAs. These noncoding RNAs play critical roles in regulating gene expression at multiple levels, including transcriptional regulation, post-transcriptional processing of RNAs, and the translation of mRNAs into proteins [2,3]. The noncoding RNAs include ribosomal RNA (rRNA), long noncoding RNA (lncRNA), small nuclear RNA (snRNA), transfer RNA (tRNA), circular RNA (circRNA), and micro-RNA (miRNA) [3]. Although the protein-coding pre-mRNAs, as well as the noncoding RNAs, undergo post-transcriptional processing to generate mature transcripts, the primary focus of this article is the detection of mRNA transcript variants.

The mammalian genome contains an estimated 20,000–30,000 protein-coding genes [4]. However, 95% of human genes undergo alternative splicing, producing an average of three mature mRNA variants per gene [5]. In addition, alternative transcription start sites (TSSs) and alternative transcription termination/polyadenylation (APA) increase the diversity in mRNA transcript variants [6]. As a result, the number of different mature mRNAs is estimated to be 60,000–90,000, outnumbering the genes from which they are derived [6,7]. Different proteins can be encoded by different transcript variants. The number of proteins that potentially can be encoded by mRNA transcripts is significantly higher, estimated to be in the range of 80,000 to 120,000 [8,9].

Gene expression studies remain fundamental to understanding gene regulation and cellular functions [10]. Despite their potential biological significance, the functional roles of transcript variants derived from gene expression analyses are often overlooked. Disregarding the analyses of transcript variants can pose a significant limitation in quantifying their expression, which is essential for gaining information about complex biological processes. The expression profiles of such variants give a measure of protein variations and protein expression, allowing for the studies of protein functions [9].

## 2. Importance of Detecting mRNA Transcript Variants

**Figure 1:**
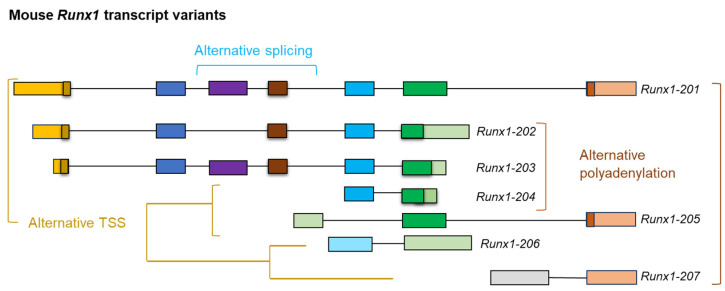
Expression of mRNA transcript variants. A schematic diagram showing the transcript variants of mouse *Runx1*. The transcript variants were expressed due to alternative transcription start sites (TSSs) in all the variants (201 to 207), alternative splicing (variant 202), and alternative polyadenylation sites (variants 202, 203, 204, and 206). This figure has been adapted from the mouse *Runx1* transcript variant map on the ENSEMBL website (not to scale).

A common misconception in gene expression analyses is the oversimplified notion that each gene corresponds to a single mRNA transcript, which, in turn, is used to produce a single protein [11]. This perspective is reductive and biologically inaccurate, especially in the context of mammalian genes that can generate multiple transcript variants [12]. The primary mechanisms responsible for increasing the diversity in mRNAs include the initiation of transcription from alternative start sites, alternative splicing, and APA [6]. These variants can differ significantly in several aspects, including the structure of their 5′-ends, the composition of their exons, and their 3′-ends [13,14,15]. Alternative start sites increase variability at the 5′-end of the transcript and may encode proteins with diverse amino termini, and APA alters the 3′-end of mRNA and generates protein diversity at the carboxy termini [16,17]. However, alternative splicing can impact any part of the coding sequences. For example, the mouse *Runx1* gene expresses transcript variants with different TSSs, APA sites, and alternative splicing [18] (Figure 1). It is also well-known that the pre-mRNAs expressed from different members of the human *BCL2* family undergo alternative splicing to form multiple isoforms [19]. While *BCL2* forms *BCL-2α* and *BCL-2β*, *BCL-X* (*BCL2L1*) forms *BCL-X_L_* and *BCL-X_S_*, and *MCL-1* forms *MCL-1L*, *MCL-1S*, and *MCL-1ES*. In addition, both *BCL-W* (*BCL2L2*) and *BFL-1/A1* form two isoforms due to alternative splicing [19,20].

While some transcript variants expressed from a gene can translate into different proteins, others may form noncoding regulatory RNAs. Although the protein-coding open reading frames (ORFs) often remain intact in transcript variants expressed by the same gene, proteins with different amino acid sequences can also be encoded by variants that gain a frameshift or isoform-specific unique sequences [21,22]. Mature mRNAs that lose their ORFs and are not translated into functional proteins may undergo decay or act as noncoding RNAs [23]. Thus, some variants may possess a more critical role than other transcript variants. The traditionally focused transcript variant approach overlooks the expression and function of remaining transcript variants. Accordingly, detecting all the transcript variants remains fundamental to understanding cell-type-specific gene regulation and cellular phenotypes.

The generation of transcript variants can be cell-type specific and linked to developmental stages or disease conditions [24]. The same gene may express different transcript variants in different tissues. The same cell lineage may also express different transcript variants from a single gene at different differentiation or developmental stages. Therefore, analyzing transcript switching is crucial for understanding cell differentiation and cell fate determination. Moreover, mutations or disease conditions may lead to the expression of different transcript variants from a single gene in the same cell type. Recent studies have suggested that altered mRNA transcript variants may be involved in disease pathogenesis, including carcinogenesis [25]. Thus, identifying the disease-specific transcript variants may serve as biomarkers for disease diagnosis and provide a potential target for drug delivery or therapeutic measures. It has been suggested that mutations may impact pre-mRNA splicing and cause diseases [26]. For example, hypercholesterolemia may result from mutated exon sequences of LDL receptors caused by the dysregulation of alternative splicing [27]. Although alternative TSSs, alternative splicing, and APA allow for the formation of functionally diverse transcript variants, they have also been reported in different types of cancers; detecting these variants and examining the underlying mechanisms are emerging fields of cancer biology and should become focal points to the challenges of cancer prevention [28].

## 3. Detection of Transcript Variants

Transcript variants expressed by different genes were identified long before genome-wide approaches were developed [29,30]. Cloning and sequencing of mRNA libraries, Northern blotting, microarrays, RNase protection assays, RACE-PCR, ddPCR, and RT-PCR have made notable contributions in identifying transcript variants. However, these techniques are suitable for selected genes, not whole transcriptome studies [31]. Recent advancements in RNA sequencing (RNA-Seq) techniques have allowed the scientific community to identify transcript variants and analyze their development mechanism and potential role in cellular functions. In the following sections, we discuss RNA-Seq in detail, followed by the abovementioned techniques that can be used to verify selected transcript variants.

### 3.1. RNA Sequencing

**Figure 2:**
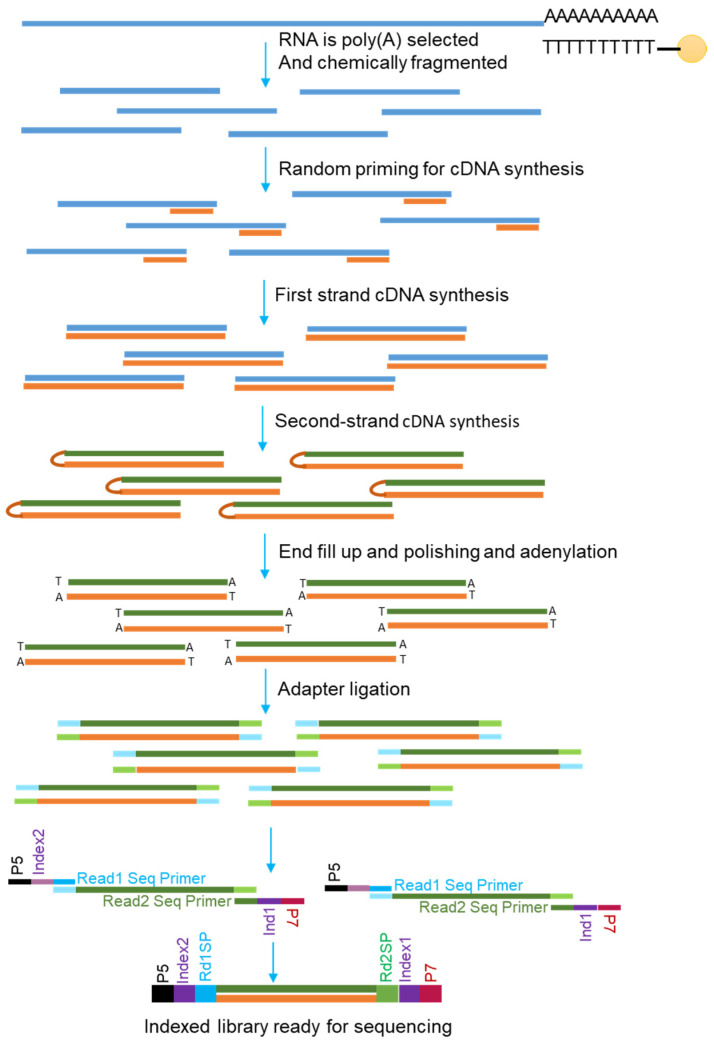
Overview of short-read mRNA sequencing. A schematic presentation of stranded mRNA library preparation using an Illumina kit. The process begins with mRNA enrichment via poly(A) selection and fragmentation. This is followed by first- and second-strand cDNA synthesis. The resulting double-stranded cDNA undergoes end repair and adenylation before ligating indexed adapters. The library is then amplified and cleaned. The final product is an indexed library ready for Illumina sequencing, containing elements like P5/P7 sequences and sequencing primer binding sites.

RNA-Seq can detect and analyze the sequences of RNA molecules present in a test sample [32]. This technique can elucidate the complexity of transcription and post-transcriptional processing of pre-mRNAs that form mature mRNAs. The first step of RNA-Seq is assessing the RNA quality using an Agilent Bioanalyzer or similar techniques. Then, high-quality total RNA (e.g., RIN value ≥ 8) or purified mRNA are reverse transcribed using oligo(dT) primers [33]. RNA-seq can also be performed with random primers, especially for transcript detection with short-read methods. Purified mRNAs are chemically fragmented for short-read sequencing, and then cDNAs are prepared. After the preparation of cDNA libraries (single- or double-stranded), adapters are ligated to either the intact (for long-read sequencing) or fragmented cDNAs (for short-read sequencing). Then, the libraries can be sequenced on strategy-specific platforms directly or after PCR amplifications (Figure 2).

The results are demultiplexed according to test samples and processed for alignment, assembly, and further analysis. In an RNA-seq experiment, identification of the alternative TSSs, alternative splicing, and APA depends on the quality of RNA, library preparation, sequencing platform, and data analysis [34]. Transcript variant analysis can be improved with paired-end sequencing due to the heavy constraints on the distance between reads when mapping along the reference sequence [35]. The counted reads aligning with each transcript quantify the transcript expression normalized by the transcript length. This process of paired-end sequencing allows for better differentiation amongst currently known variants and a total quantification of reads [35].

Recent advances in RNA-Seq technology have expanded its applications in various experimental settings. Illumina (San Diego, CA, USA), Oxford Nanopore Technologies (ONT, Oxford, UK), and Pacific Biosciences (PacBio, Menlo Park, CA, USA) are the most popular RNA-Seq methods [36]. ONT and PacBio can perform long-read sequencing; however, ONT uniquely enables the direct sequencing of full-length RNA molecules without converting them to cDNA. This allows ONT to detect RNA modifications, such as methylation, alongside nucleotide sequences [37]. In contrast, PacBio relies on cDNA-based methods for RNA sequencing and cannot directly sequence native RNA molecules [38]. Other sequencing methods include single-cell RNA sequencing (scRNA-Seq) using 10x Genomics (Pleasanton, CA, USA), Takara Bio (SMART-Seq) (San Jose, CA, USA), or Bio-Rad (ddSEQ, Hercules, CA, USA) systems to render various cell libraries at once and view the cellular landscape of tissues. The scRNA-seq libraries are run on a standard sequencing platform (Illumina, ONT, or PacBio), and the sequencing data are analyzed using specific software.

Bulk RNA-Seq (e.g., Illumina, ONT, and PacBio) uses RNA samples to provide gene expression profiles across tissue or a population of cells that may contain more than one cell type [39]. Despite the cost-effectiveness of bulk sequencing, it represents a mixed expression profile of all the cell types present in the tissue. This process fails to distinguish genes expressed in specific cell types and captures cellular heterogeneity [40]. In contrast, scRNA-Seq (e.g., 10x Genomics) allows for the detection of cellular heterogeneity and identification of individual cell populations while also giving gene expression profiles for the libraries it creates [41,42]. These expression profiles can be analyzed via a standard RNA-seq platform. However, the gene expression data at the single-cell level come with a few limitations. In addition to the higher cost and computational complexity, obtaining fresh cells by removing dead cells and cell debris remains a limitation to generating precise transcriptome data [40,43].

#### 3.1.1. Short-Read Versus Long-Read mRNA Sequencing

Short-read mRNA sequencing (e.g., Illumina) involves isolating poly-A mRNA using oligo(dT) magnetic beads, followed by the fragmentation of mRNAs and reverse transcription into first-strand cDNA with random primers. Strand specificity is achieved by incorporating dUTP in the second strand, which is later degraded. Adapters are ligated to the fragments, and the library is amplified via PCR (Figure 2). Sequencing can be performed using Illumina’s sequencing-by-synthesis (SBS) technology, offering accurate transcriptome analysis [44,45].

In contrast to short-read mRNA sequencing, long-read mRNA is a powerful technique that allows reading the full-length RNA molecules without fragmentation [46]. This method identifies the sequences of full-length mRNA transcripts, which can be analyzed to determine alternative TSSs, splicing events, APA, gene mutations, and gene fusions accurately [47]. ONT offers two methods for long-read mRNA sequencing: direct RNA and cDNA-based. Direct RNA sequencing isolates poly-A RNA using poly-T oligo beads, ligates adapters directly to native RNA without reverse transcription, and sequences it through nanopores, preserving RNA modifications and strand specificity [48,49]. Although ONT’s direct RNA sequencing takes steps to avoid most biases, the poly-A selected RNA populations introduce a capture bias that could be misinterpreted as differential gene expression [50]. This 3′ bias occurs when the poly-A selection skews the sequenced mRNAs toward the lengthier tails, creating a distorted view of the transcriptome. It is best to omit the selection during pre-processing when sequencing with a poly-A selection RNA population [50]. On the other hand, cDNA-based sequencing involves the reverse transcription of poly-A RNA into full-length cDNA, followed by adapter ligation and amplification, providing higher throughput but losing RNA modification information [32].

PacBio follows a similar cDNA-based approach to sequencing with the current Iso-Seq technique where cDNA is synthesized; random primers are annealed for first-strand synthesis, and reverse transcription activates template switching, followed by cDNA amplification and adapter ligation. These sequenced full-length transcript reads have a maximum insertion size of 10 kilobase pairs [51]. Circular consensus reads, such as HiFi reads, can improve accuracy for isoform detection and transcriptome annotation when combined in the same pipeline with Iso-Seq. Overall, short-read sequencing is typically highly accurate and ideal for gene expression profiling and SNP detection but struggles to resolve full-length transcripts or complex genomic regions due to its short read lengths (50–300 bp) [52,53]. Long-read sequencing captures full-length transcripts, resolves structural variants, and detects isoforms.

#### 3.1.2. Direct Versus PCR-Amplified Detection of mRNAs

Direct mRNA sequencing (dRNA-Seq) allows the direct detection of full-length mRNA transcripts and the characterization of RNA modifications [48]. After purifying the mRNAs from total RNAs and assessing their quality, dRNA-Seq can be performed using ONT technology. Libraries are prepared using oligo(dT) primers, and first-strand cDNA is synthesized by reverse transcription [54]. Then, the mRNA-cDNA hybrid is purified, and the sequencing adapters are ligated, purified again, and loaded onto platform-specific Flow Cells to run for sequencing. The protocol for cap-dependent ligation includes dephosphorylating and de-capping mRNA and ligating a biotinylated 5′ adapter RNA [55]. After purifying and reassessing, processing is carried out with the library preparation, as described [56]. This method provides comprehensive insights into RNA molecules in their native form [48].

PCR-amplified RNA sequencing involves increasing the quantity of target cDNA libraries using 5 to 10 cycles of PCR. The PCR primers can bind to the adapters ligated to cDNA ends. The cDNA library is amplified to generate adequate cDNA material for sequencing. After purifying the PCR-amplified cDNA library, the final step is to sequence on an appropriate platform [57]. There are some potential drawbacks to the PCR amplification of RNA-Seq libraries [32]. PCR amplification of the cDNA sequences is not linear and introduces amplification bias due to primer bias, GC content bias, and secondary structure bias [58,59]. Amplification bias may also result in over-representation of the abundant transcripts and sequencing errors due to base misincorporation [60,61]. Recent studies have suggested incorporating unique molecular identifiers (UMIs), the optimization of PCR conditions, and low-cycle PCR to mitigate PCR amplification bias [61,62,63].

#### 3.1.3. Bulk Sequencing Versus Single-Cell Sequencing of mRNAs

Bulk RNA-seq is a method for analyzing the whole transcriptome of a sample by sequencing the mRNAs irrespective of cellular origin. Bulk sequencing is performed using either a short-read or long-read strategy. After converting the intact or fragmented mRNAs to cDNA, sequencing adapters are ligated. Then, the cDNA libraries are processed with or without PCR amplification and run on a specific sequencing platform. The sequencing data output is demultiplexed according to the adapter sequences and analyzed using different commercial or open software.

Single-cell mRNA sequencing can be performed using flow sorting (Takara Bio USA, San Jose, CA, USA) or microfluidics methods (10x Genomics). Takara Bio’s SMART-Seq mRNA Single-Cell LP protocol is optimized for generating high-quality Illumina-ready libraries from single cells, especially those with a low RNA content. It uses SMART (Switching Mechanism at the 5′-end of RNA Template) technology, which employs a template-switching reverse transcriptase to enrich full-length cDNA and add PCR adapters directly to both ends. This ligation-free workflow minimizes handling errors and accurately represents mRNA transcripts, including the 5′-ends. The protocol involves enzymatic fragmentation, stem–loop adapter ligation, and library amplification, producing sequencing-ready libraries within two days [64]. For 10x Genomics, cells move through channels within Chromium X instruments at a limited dilution and generate nanometer-sized gel beads-in-emulsion (GEMs). This technique uses microfluidic partitioning to encapsulate single cells in GEMs containing barcoded oligonucleotides. Reverse transcription occurs within GEMs, attaching UMIs and cell barcodes to cDNA. After breaking the emulsion, cDNA is amplified, fragmented, and prepared into sequencing-ready libraries compatible with Illumina platforms. This method enables the high-throughput processing of thousands of cells in a single run, capturing 3′ gene expression profiles with high resolution. Data analysis is performed using the Cell Ranger pipeline for cell-specific transcriptome mapping [65,66].

Single-cell mRNA sequencing, such as Takara Bio’s SMART-Seq and 10x Genomics, captures cellular heterogeneity and rare populations and is valuable for studying cell types in complex tissues, such as the brain or immune system, where understanding cellular differences is crucial [67]. Takara excels in full-length transcript analysis using SMART technology, while 10x enables high-throughput profiling of thousands of cells via microfluidic barcoding methods [68,69]. Consequentially, due to the low capture efficiency of isoforms by scRNA-Seq overall, forced detection of splicing events results in confounding [70]. Since the amount of single-cell material initiating library preparation is too small, the frequency of dropouts in splicing analysis becomes apparent. This limitation will remain unless the preparation for scRNA-Seq changes, which poses a significant challenge. Hence, it is recommended not to attempt large-scale alternative splicing analysis during scRNA-Seq. Bulk RNA-seq, by contrast, measures average gene expression across cell populations, making it cost-effective for global transcriptome analysis but unable to resolve individual cell types or rare populations [40,71].

#### 3.1.4. Analysis of RNA Sequencing Data

Data analysis begins with importing the RNA-Seq data to the pipeline and removing the adapter sequences from cDNA libraries [72]. For the short-read sequence analysis, each cDNA fragment sequence is read through a commercial data-processing platform, such as CLC Genomics (Qiagen Bioinformatic), Partek Flow (Illumina), or DNASTAR Lasergene (DNASTAR, Madison, WI, USA). After reading the nucleotide sequences of the cDNA fragments, the fragments are assembled and aligned to a reference genome to ensure accurate mapping of each read to the correct location. These aligned reads are quantified to determine gene expression levels, involving counting and normalizing the reads corresponding to each gene [73]. Data alignment against a reference genome identifies the splice junctions and denotes the alternatively spliced variants [74].

**Figure 3:**
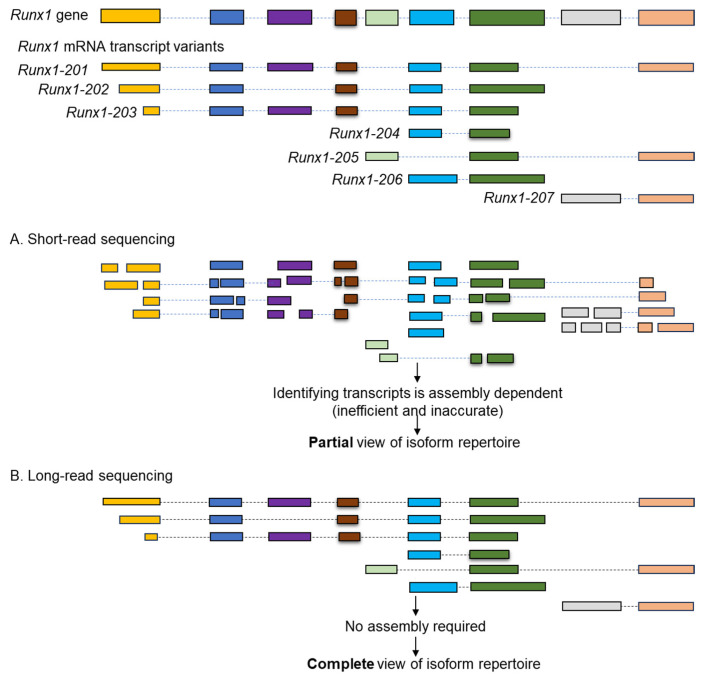
Comparison of short-read and long-read RNA-seq data analysis. A schematic presentation of *Runx1* transcript assembly and variant detection using short-read (**A**) and long-read (**B**) sequencing. Short-read sequencing requires assembly, leading to an inefficient and potentially inaccurate view of the isoform repertoire. In contrast, long-read sequencing, with its ability to sequence full-length transcripts, eliminates the need for assembly and provides a complete view of the *Runx1* isoform repertoire.

After such alignments, gene and transcript level quantifications are undertaken by normalizing with the total read counts (e.g., TPM, RPKM, etc.). Gene-level quantification assigns all the cDNA fragments to a gene, where all transcripts are aligned to that gene locus [75]. Generally, the gene expression (GE) values represent the sum of all expressions from the transcript variants expressed from that gene [76,77]. Transcript-level quantification assigns cDNA fragments to the reference transcript sequences. Although the fragments may be ambiguous, alignment with the reference transcripts allows for superior biological resolution, giving insight into isoform switching, which is overlooked during gene-level quantification [78]. Once the assembly, alignment, and quantification are completed, differential expression analysis can be employed to understand the regulation of gene or transcript expression. Short-read RNA-Seq data face difficulty identifying transcript variants due to their limited read length. These include difficulties in mapping reads uniquely to specific isoforms, resolving complex splicing events, and handling multi-mapped reads in repetitive or homologous regions and biases in quantification methods like TPM or RPKM that may not entirely correct for sequencing artifacts [79,80] (Figure 3).

Unlike their long-read counterparts, more pipelines and open software are available for short reads, such as the Illumina data set. However, long-read sequencing technologies can still enable precise identification of transcript isoforms by directly sequencing full-length transcripts and capturing exon–intron structures and alternative splicing patterns without assembly. These methods provide detailed isoform-specific quantification, overcoming the limitations of short-read sequencing [81]. Challenges such as higher raw error rates necessitate robust error correction tools like Minimap2, assembled by Canu or Miniasm, and Iso-Seq pipelines. These ensure accuracy while addressing small exon alignment and splice site prediction [51].

### 3.2. Hybridization-Based Techniques

#### 3.2.1. Spatial Transcriptomics

Spatial transcriptomics is an advanced technique that allows researchers to map gene expression within the context of tissue architecture [82,83]. It begins with the preparation of tissue sections to maintain their structure [84]. Specific probes are introduced to target mRNA molecules within the tissue. The probes that bind to target mRNAs are detected using imaging or sequencing techniques [85]. This enables the detection of RNA, aiding in identifying cell types based on their gene expression [86]. The in situ sequencing method uses padlock probes and rolling circle amplification to target and amplify specific RNA molecules within preserved tissue sections [87,88]. The SCRINSHOT approach hybridizes padlock probes on mRNA, followed by circularization and rolling circle amplification [89]. This method allows for the multiplexed detection of thousands of cells in tissue sections, providing a detailed map of cell states and gene expression [89]. Despite being a powerful tool for understanding cellular heterogeneity and spatial organization in tissues, not all spatial transcriptomics can achieve high cellular resolution. Overall limitations in the sequencing-based approach resolution come from the physical size of the capturing spot since multiple cells need to be captured [90]. Imaged-based methods are more suitable for single-cell or subcellular resolution but have the sole limitation of their optical diffraction limit [91,92]. Both spatial transcriptomic techniques can be used for their specific strengths, but efforts to refine their sensitivity and cellular resolution are ongoing. As spatial transcriptomics evolves, the ability to visualize RNA molecules in their home environment will allow for the accurate identification of novel transcript variants in a cell type within a tissue section [93].

#### 3.2.2. Microarrays

Microarrays, also known as gene chips, are powerful tools for simultaneously studying gene expression patterns across a vast number of genes [94]. The process begins with the extraction of mRNA from the sample of interest. This mRNA is then converted to cDNA and labeled with fluorescent markers. The labeled cDNAs are hybridized to DNA probes on the microarray chips, which contain thousands of probes designed to hybridize with specific mRNA molecules. After hybridization, the chip is scanned to detect fluorescent signals [95]. These signals indicate the presence and abundance of specific mRNA molecules in the test sample. Depending on the probe sequences, mRNA transcript variants expressed in a particular sample can be detected [95]. The detection of transcript variants will depend on the probe design; if the target exon is absent in a variant, it will remain undetected. Polymorphisms or mutations also remain undetected.

#### 3.2.3. Northern Blotting

Northern blotting is the standard method for detecting RNA expressions in particular tissues or cell types [96]. Denatured total RNA is separated on agarose gels by electrophoresis and transferred to nitrocellulose or nylon membranes by the capillary method [97]. After cross-linking the RNA to the membranes by UV exposure, the membranes are hybridized to radioactive or non-radioactively labeled RNA or DNA probes [96]. Signals from the bound probes can be imaged by autoradiography or automated imaging systems [97]. Signals from bands with different molecular weight sizes indicate the presence of multiple mRNA transcript variants. However, Northern blotting suffers from several limitations. The detection of the transcript variants depends on the probe targets. The resolution of Northern blotting is not high enough to differentiate transcripts with similar lengths [98]. Moreover, Northern blots can detect only a limited number of genes and cannot be used for the detection of novel genes.

#### 3.2.4. RNase Protection Assays

The RNase protection assay is a sensitive technique that aims to identify and measure the abundance of specific mRNAs [99]. This technique utilizes a procedure that hybridizes RNA with a radioactively labeled RNA probe designed for a particular mRNA [100]. To design this probe, a specific DNA template is needed for in vitro transcription to synthesize an antisense RNA [101]. During the synthesis, one of the four nucleotides is replaced with a corresponding radioactively labeled nucleotide to allow for the identification of the probe during later steps. After the antisense RNA probe is synthesized, the DNA template is removed through DNase digestion. Once the probe is purified, the isolated RNA is mixed with the labeled probe for hybridization. Then, the RNA–prehybridization mix is treated with RNase A/RNase TI and purified with a phenol–chloroform mix. While hybridization protects the targeted mRNA-probe heterodimer during digestion, anything not hybridized with the probe remains unprotected [102]. After digestion, the reaction is precipitated, gel electrophoresed, and detected by autoradiography [103]. This technique can detect the presence and abundance of a specific transcript variant, but it is not applicable to genome-wide applications.

### 3.3. PCR-Based Techniques

#### 3.3.1. RACE PCR

Rapid Amplification of cDNA Ends (RACE PCR) is a technique that is used to determine the full-length sequence of an mRNA as long as part of the transcript sequence is known [104]. RACE PCR is efficient and inexpensive; it requires a cDNA synthesis and a single PCR reaction to amplify the desired 5′- or 3′-end of a certain cDNA of interest. Two types of RACE PCR are in use: 5′ RACE or 3′ RACE. The first step for both procedures is to utilize reverse transcription to create a cDNA copy of a region of RNA transcript [105].

The 5′ RACE approaches are slightly more complex than the 3′ RACE since the 5′-ends on the mRNAs do not have generic priming sites, as seen in the poly(A) tail [106]. The addition of an adapter sequence at the 5′-end will lead to accurate characterizations of the 5′ UTR and completion of the 5′ RACE. Current methodologies take advantage of the MMLV reverse transcriptase during first-stand cDNA synthesis [107]. Typically, 2–4 non-templated cytosine residues are added to the 3′-end of synthesized cDNAs once the mRNA reaches the 5′-end, cap region. Terminal 3–4 G residues base pairs with 2–4 C residues of the created cDNA if an oligonucleotide with oligo(G) or oligo(rG) sequences is included in the incubation medium [105]. The new template will elicit a reverse transcriptase template switch and replicate the sequence of the oligonucleotide [105]. As the MMLV reverse transcriptase adds C residues to the cDNA, whole cDNA libraries are amplified with full-length clones. A homopolymer tail is then joined to the cDNA end using terminal deoxynucleotidyl transferase, which can bind a universal primer during downstream PCR [108]. Then, PCR is performed using a reverse gene-specific primer and a universal primer (UAP1). These primers amplify cDNA, generating a product that includes the unknown 5′ sequence.

In 3′ RACE, an oligo-dT-containing primer complementary to the poly(A) tail (including an adapter oligo sequence) is used to bind to mRNA transcripts and reverse transcribed to synthesize the 3′-end of the cDNA [109]. Then, PCR is performed using a gene-specific forward primer that binds to a known cDNA sequence and a primer that binds to the adapter sequence [110]. This process produces a product that includes the sequence of interest at the 3′-end. After the initial cycle of PCR for both 5′ RACE and 3′ RACE, additional PCR cycles are carried out using nested primers to increase specificity [111]. In both cases, a known section of a specific mRNA transcript is used to create a cDNA of the entire transcript sequence, which is then amplified during PCR. Thus, this process can detect the unknown sequences of a single mRNA transcript variant.

#### 3.3.2. RT-PCR and RT-qPCR

**Figure 4:**
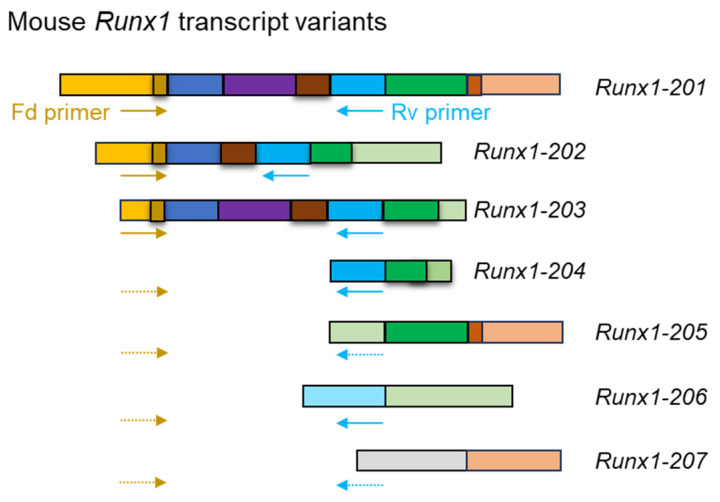
Detection of mRNA transcript variants using RT-PCR. A schematic illustrates the exon composition of the *Runx1* transcript variants. RT-PCR can be performed using primers designed for variant-specific exons. Using the forward (Fd) and reverse (Rv) primers, *Runx1-201*, *202*, and *203* can be detected, but the remaining variants cannot be detected due to the failure of primer binding. Moreover, RT-PCR will fail to differentiate Runx1-201 and Runx1-203. Solid arrows indicate the binding of primers to target templates, whereas dotted arrows indicate an inability to bind.

The first step of the reverse transcription polymerase chain reaction (RT-PCR) is the reverse transcription of mRNA to generate cDNA. cDNA is used as a template for the downstream PCR reaction to amplify the cDNA sequences. Amplified PCR products can be visualized through gel electrophoresis, followed by ethidium bromide or SYBR Green staining. RT-PCR is a widely used method to determine the presence of specific RNA segments, leading to discoveries in gene expression. This method can also be applied to detect transcript variants; specific primers can be designed to bind to variant-specific exons or regions, flanking alternatively spliced regions to amplify the presence of a specific variant (Figure 4). This allows researchers to identify which variants are present in the sample and further our understanding of the variants’ functional diversity.

RT-qPCR is a process like RT-PCR, but its primary purpose is quantifying cDNAs. The procedure for RT-qPCR fluorescent dyes or probes monitors the accumulation of the PCR product in real time. This process easily allows the quantifying of specific cDNAs. It can also be applied to detect transcript variants, as it can quantify levels of different transcript variants. Using specific primers or probes unique to each variant, researchers can measure the relative abundance of each variant in a sample. As RT-PCR and RT-qPCR can efficiently detect selective mRNA transcript variants, these techniques are often used to verify RNA sequencing results.

### 3.4. Machine Learning

The recent advancement in the field of variant detection comes in the form of prediction algorithms in machine learning. Studies have used splice prediction tools, although there is still inadequate consensus for a tool to be optimal. MaxEntScan (MES) is an older method that is based on a maximum entropy model to capture the highest score as the best fit for a potential donor or accept site [112]. Another sliding window algorithm called MES-SWA can be used to capture the transcript variants. Modular Modeling of Splicing (MMSplice) is a software built on multiple neural networks and deep learning frameworks trained to score exon, intron, and other splice sites to predict transcript variants [113]. Super Quick Information-content Random-forest Learning of Splice variants (SQUIRLS) stands out as a unique approach to interpreting nucleotide changes at the 3′ and 5′ UTRs as it generates interpretable features through its engineered decision trees to classify splice sites [113,114]. SpliceAI is another AI tool that was trained initially based on human reference genome data and used for the identification of splice junctions [115,116]. It uses neural networks that work on 10,000 nucleotides of context to predict splice sites. A collapsed isoform (CI) set representing manually annotated constitutive and alternative splice sites was included in SpliceAI to improve prediction. The CI-SpliceAI gained more accuracy in predictions [115,117,118]. ASTK, a recent software package that covers both the downstream and upstream analysis of alternative splicing, provides enrichment analysis at the gene and exon levels. This package extracts the specific features of the targeted sequence, as well as its epigenetic marks that align with splicing events, uncovering that splice strength is a determinant for A3 and A5 exon inclusion levels [119].

Methods that enhance machine learning algorithms for a better understanding of splice variant function and alternative splicing events are still improving [120]. The increased ability to locate the motifs of RNA-binding proteins and their splicing effects will improve the detection of alternative splicing. Including newer elements, such as epigenetic marks, in these algorithms will also play an essential role in discovering the intricacies of splice regulation.

## 4. Advantages and Disadvantages of Detection Methods

The applicability of any detection technique will depend on the efficiency of sequencing the whole mRNA from the 5′-end to the 3′-end of mRNAs. Considering the available techniques, RNA-Seq is the best for detecting mRNA transcript variants. Illumina, PacBio, and Nanopore technologies are the most popular RNA-Seq techniques [36]. Other sequencing methods include scRNA-Seq using 10x Genomics, Takara Bio, or Bio-Rad systems. Illumina sequencing is the most accurate and cost-effective NGS platform, making it suitable for research. However, despite its high accuracy, RNA-Seq on the Illumina platform suffers from de novo assembly and alignment problems of the short reads. In this regard, long-read sequences on nanopore or PacBio appear better. The key advantage of nanopore or PacBio sequencing is its ability to perform long reads up to hundreds of kilobases. These techniques can sequence RNAs without PCR amplification, reducing PCR biases and errors. However, it must be noted that the random error rate of base reading in nanopore or PacBio (5–15%) is remarkably higher than that of Illumina (0.1–0.5%) [121]. The initial establishment cost of the Illumina or PacBio system is much higher than that of nanopore technologies. However, the sequencing cost per sample is much higher in nanopore or PacBio systems. Newer high-throughput sequencing systems and reagent chemistry have lowered the cost of nanopore sequencing, which is still higher than Illumina systems [122].

Direct RNA-Seq using nanopore technology remains the best laboratory technique for detecting mRNA transcript variants. However, these bulk RNA sequences can represent a tissue or organ but cannot identify the cellular origin of specific transcript variants. scRNA-Seq can identify the cellular origin of the transcript, but commonly used scRNA-Seq (e.g., 10x Genomics), which targets the 3′-end of mRNA only, is not useful for analyzing transcript variants. However, mRNA sequencing of isolated single cells (Takara bio or low-input RNA-seq) or full-length RNA-Seq in single cells (using combined nanopore and 10x Genomics) is applicable for mRNA transcript variant detection. However, these methods may exhibit a low depth of RNA sequencing.

Another limitation remains with analyzing RNA-Seq data. RNA-Seq performed on the Illumina platform can be analyzed using many open or commercial software programs. In contrast, only a limited number of software programs are available to analyze RNA-Seq data from other platforms. Despite the labor-intensive procedures, hybridization-based methods are not as efficient as RNA-Seq methods in detecting mRNA sequence variants [123]. However, PCR-based methods have selective advantages and can complement the RNA sequencing approach [32]. RT-PCR and Sanger sequencing can be used to verify any RNA sequencing data.

## 5. Conclusions and Future Perspectives

Only one mRNA transcript expressed from a gene is often focused on in gene expression studies. However, this simplistic approach is biologically inaccurate; most mammalian genes express more than one mature mRNA. The major barrier in transcript variant analysis is the efficacy and cost-effectiveness of the detection methods. Considering the available laboratory techniques, RNA-Seq is the best for detecting mRNA transcript variants. Compared to short-read-based sequencing using the Illumina platform, long-read sequences on ONT or PacBio platforms appear better due to de novo assembly and alignment advantages. However, long-read sequences and direct mRNA sequencing require large quantities of RNA, which is feasible for bulk sequencing. Bulk sequencing with long reads can identify mRNA transcript variants efficiently, but it fails to determine the cellular origin of a particular transcript variant. scRNA-Seq can resolve the issue of cell type identification. However, scRNA-Seq typically detects the 3′-end of mRNAs. Researchers have addressed this issue by combining ONT or PacBio sequencing (long read) with 10x Genomics-based scRNA-Seq. We anticipate that future studies will be directed toward developing scRNA-Seq techniques that perform long-read direct mRNA sequencing.
